# Quantitative evaluation of emphysema for predicting immunotherapy response in patients with advanced non-small-cell lung cancer

**DOI:** 10.1038/s41598-022-13131-2

**Published:** 2022-05-25

**Authors:** Yoshimi Noda, Takayuki Shiroyama, Kentaro Masuhiro, Saori Amiya, Takatoshi Enomoto, Yuichi Adachi, Reina Hara, Takayuki Niitsu, Yujiro Naito, Kotaro Miyake, Shohei Koyama, Haruhiko Hirata, Izumi Nagatomo, Yoshito Takeda, Atsushi Kumanogoh

**Affiliations:** 1 Department of Respiratory Medicine and Clinical Immunology, Osaka University Graduate School of Medicine, 2-2 Yamadaoka, Suita City, Osaka 565-0871 Japan; 2 Department of Immunopathology, WPI, Immunology Frontier Research Center (iFReC), Osaka University, Suita, Osaka Japan; 3 Integrated Frontier Research for Medical Science Division, Institute for Open and Transdisciplinary Research Initiatives (OTRI), Osaka University, Suita, Osaka Japan; 4 Center for Infectious Diseases for Education and Research (CiDER), Osaka University, Suita, Osaka Japan

**Keywords:** Cancer, Immunology, Medical research, Oncology

## Abstract

The efficacy of immune checkpoint inhibitors (ICIs) in patients with advanced non-small-cell lung cancer (NSCLC) might depend on the presence of emphysema, but this association is not established. We aimed to investigate if quantitively and automatically measuring emphysema can predict the effect of ICIs. We retrospectively analyzed 56 patients with NSCLC who underwent immunotherapy at our hospital. We used the Goddard scoring system (GS) to evaluate the severity of emphysema on baseline CT scans using three-dimensional image analysis software. The emphysema group (GS ≥ 1) showed better progression-free survival (PFS) than the non-emphysema group (GS = 0) (6.5 vs. 2.3 months, respectively, p < 0.01). Multivariate analyses revealed that good performance status, GS of ≥ 1, and high expression of PD-L1 were independently associated with better PFS, while smoking status was not. In conclusion, quantitative evaluation of emphysema can be an objective parameter for predicting the therapeutic effects of ICIs in patients with NSCLC. Our findings can be used to generate hypotheses for future studies.

## Introduction

Immune checkpoint inhibitors have improved clinical outcomes in patients with advanced lung cancer. However, their therapeutic benefits are limited to a few patients, and the perfect predictors of these benefits remain unknown. The expression of PD-L1 and tumor mutational burden (TMB) are some tumor-related factors that might be responsible for enhancing the effects of immunotherapy, but they are imperfect. Therefore, there is a need to comprehensively evaluate multiple factors that may affect the action of ICIs in cancer patients.

Among host factors, smoking status, which increases somatic mutations and expresses neoantigens, is beneficial for ICIs in patients with advanced non-small-cell lung cancer (NSCLC)^1,2^. However, investigating the patients’ smoking status by conducting interviews does not necessarily reflect the actual smoking exposure or its mutational effect^3^. A previous meta-analysis showed that emphysema detected on computed tomography (CT) was independently associated with an increased risk of lung cancer irrespective of smoking status^4^. The study also indicated that a shared mechanism might be involved in the pathogenesis of both emphysema and lung cancer.

However, despite this evidence, the association between treatment outcomes of ICIs and the development of emphysema has not been investigated. Therefore, we aimed to evaluate whether the quantitative evaluation of semiautomatically detected pulmonary emphysema can predict the effectiveness of ICI treatment in patients with advanced NSCLC.

## Results

### Patient characteristics and Goddard score

We identified 56 patients with advanced NSCLC who received monotherapy with ICIs. A total of 15 patients had a Goddard score (GS) = 0, and 41 patients had a GS ≥ 1. The median body mass index (BMI) was higher in the group of GS = 0 than in the group of GS ≥ 1 (23.2 vs 21.3 kg/m^2^, p = 0.04). There were no significant differences in patient characteristics and laboratory findings other than BMI between the two groups (Table 1).

**Table 1:** Baseline patient characteristics according to the Goddard score.

	Goddard score 0 (N = 15)	Goddard score ≥ 1 (N = 41)	P-value
**Age (years)**			
Median (IQR)	72 (64–75)	70 (66–74)	> 0.99
**Sex, n (%)**			0.35
Male	7 (46.7)	13 (31.7)	
Female	8 (53.3)	28 (68.3)	
**Body mass index (kg/m**^**2**^**)**			0.04
Median (IQR)	23.2 (20.9–25.0)	21.3 (18.9–23.2)	
**ECOG PS, n (%)**			0.60
0	2 (13.3)	4 (9.8)	
1	9 (60.0)	22 (53.7)	
2	3 (20.0)	14 (34.1)	
3	1 (6.7)	1 (2.4)	
**Smoking status, n (%)**			0.67
Never	3 (20.0)	5 (12.2)	
Current or former	12 (80.0)	36 (87.8)	
No. of patients evaluated by HRCT, n (%)	14 (93.3)	37(90.2)	> 0.99
**Histology, n (%)**			0.24
Adenocarcinoma	7 (46.7)	27 (65.9)	
Squamous cell carcinoma	4 (26.7)	10 (24.4)	
NOS	4 (26.7)	4 (9.8)	
**PD-L1 TPS, n (%)**			0.50
< 1%	2 (13.3)	5 (12.2)	
1–49%	2 (13.3)	12 (29.3)	
≥ 50%	9 (60.0)	22 (53.7)	
Unknown	2 (13.3)	2 (4.9)	
**Treatment, n (%)**			> 0.99
Nivolumab	4 (26.7)	13 (31.7)	
Pembrolizumab	9 (60.0)	23 (56.1)	
Atezolizumab	2 (13.3)	5 (12.2)	
**Treatment line, n (%)**			> 0.99
1st	7 (46.7)	18 (43.9)	
≥ 2nd	8 (53.3)	23 (56.1)	
**Neutrophil-to-lymphocyte ratio**			
Median (IQR)	4.08 (3.11–5.37)	4.57 (2.84–6.76)	0.39
**Albumin (g/dL)**			
Median (IQR)	3.40 (2.95–3.75)	3.40 (2.90–3.90)	0.90
**C-reactive protein (mg/dL)**			
Median (IQR)	1.45 (0.49–4.81)	0.40 (0.11–5.16)	0.27

*IQR* inter-quartile range, *ECOG PS* Eastern Cooperative Oncology Group performance status, *HRCT* high-resolution computed tomography, *NOS* not otherwise specified, *TPS* tumor proportion score.

### Smoking history and Goddard score

**Figure 1:**
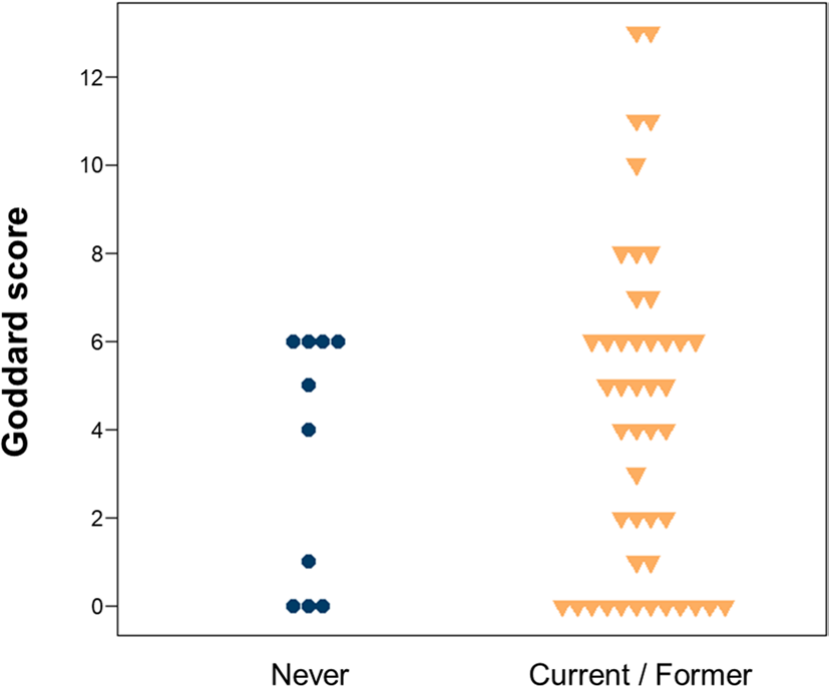
Relationship between smoking history and Goddard score.

**Figure 2:**
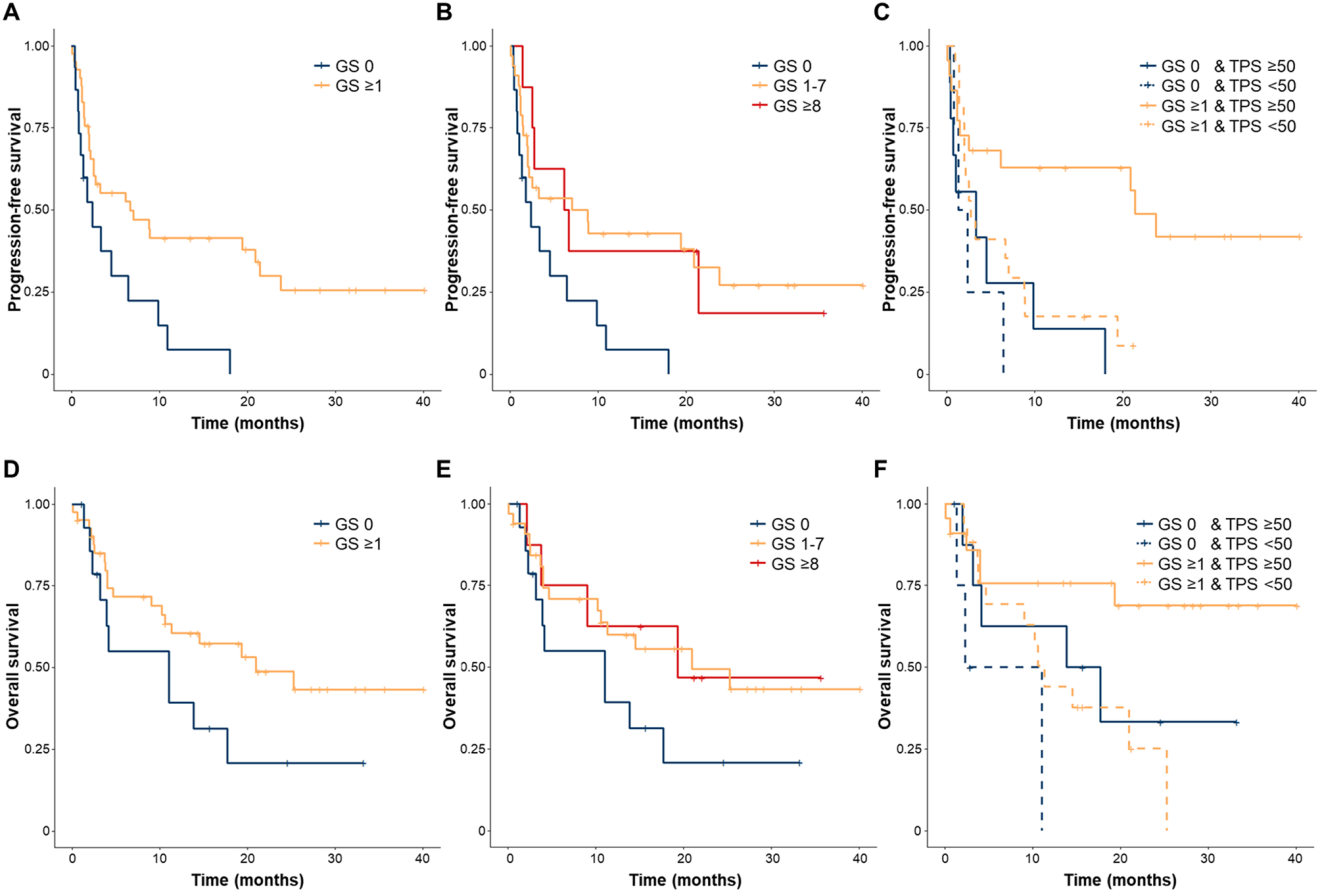
(**A**) Kaplan–Meier plot of the progression-free survival (PFS) in patients with or without emphysema (GS ≥ 1 vs GS = 0). (**B**) PFS in patients without emphysema (GS = 0), with mild emphysema (GS 1–7), or moderate emphysema (GS ≥ 8). (**C**) PFS in patients with or without emphysema, according to whether the PD-L1 expression is high or not. (**D**) Kaplan–Meier plot of the overall survival (OS) in patients with or without emphysema (GS ≥ 1 vs GS = 0). (**E**) OS in patients without emphysema (GS = 0), with mild emphysema (GS 1–7), or moderate emphysema (GS ≥ 8). (**F**) OS in patients with or without emphysema, according to whether the PD-L1 expression is high or not. *GS* Goddard score.

We analyzed the association between GS and smoking status (Fig. 1) and found no significant difference in the score between patients who had smoked and those who had never smoked (p = 0.35). Table 2 summarizes the immune responses to ICIs between the two groups. Although not statistically significant, the overall response rate was higher in the emphysema group than in the non-emphysema group (34.1% vs. 6.7%, p = 0.086). Figure 2 shows the Kaplan–Meier curves of progression-free survival (PFS) and overall survival (OS) of both groups. The median follow-up time was 11.0 months. The emphysema group showed better PFS than the non-emphysema group (6.5 vs 2.3 months, respectively, p < 0.01) (Fig. 2A). To further clarify our findings, we divided the emphysema group into two groups—the mild emphysema group (GS 1–7) and moderate emphysema group (GS ≥ 8)—but there was no statistical difference between them (Fig. 2B).

**Table 2:** Treatment outcome according to the Goddard score.

	Goddard score 0 (N = 15)	Goddard score ≥ 1 (N = 41)	P-value
**Treatment response, n (%)**			
Complete response	0 (0)	2 (4.9)	
Partial response	1 (6.7)	12 (29.3)	
Stable disease	4 (26.7)	9 (22.0)	
Progressive disease	10 (66.7)	16 (39.0)	
Not evaluable	0 (0)	2 (4.9)	
Overall response rate (95% CI)	6.7 (0.2–31.9)	34.1 (20.1–50.6)	0.086
Disease control rate (95% CI)	33.3 (11.8–61.6)	56.1 (39.7–71.5)	0.23

*CI* confidence interval.

Among patients with high PD-L1 expression, emphysema was associated with significantly better PFS as compared to no emphysema (21.1 vs 3.3 months, respectively, p < 0.01). On the other hand, among patients with low PD-L1 expression, there was no significant difference in PFS between patients with and without emphysema (2.7 vs 2.0 months, respectively, p = 0.31). There was a statistically significant difference in emphysema and PD-L1 status among the four groups (log-rank test for trend, p < 0.01) (Fig. 2C). The relationship between OS and emphysema showed a trend similar to that of PFS (Fig. 2D–F). The emphysema group showed better OS than the non-emphysema group (20.6 vs 10.8 months, respectively, p = 0.092) (Fig. 2D).

The outcomes of the univariate and multivariate analyses regarding PFS are shown in Table 3. In the univariate analyses, better PFS was significantly associated with high PD-L1 expression and emphysema (GS ≥ 1), while a treatment line of ≥ 2 was associated with worse outcomes. Multivariate analyses adjusted for age, Eastern Cooperative Oncology Group performance status (ECOG PS), BMI, smoking status, PD-L1 status, and GS revealed that emphysema (GS ≥ 1), good PS (PS ≤ 1), and high PD-L1 expression were independently associated with better PFS, while smoking status was not. Table 4 shows the findings of the univariate and multivariate analyses regarding OS. The univariate analysis demonstrated that high PD-L1 expression was the only factor related to better OS. However, after adjustment for age, ECOG PS, BMI, smoking status, PD-L1 status, and GS, multivariate analyses revealed that good PS (PS ≤ 1), high PD-L1 expression, and emphysema (GS ≥ 1) were independently associated with better OS, whereas smoking status was not.

**Table 3:** Univariate and multivariate analyses of risk factors related to progression-free survival.

Variables	Univariate analysis		Multivariate analysis	
	HR (95% CI)	P value	HR (95% CI)	P value
Age ≥ 75 years	1.03 (0.49–2.17)	0.93	1.01 (0.45–2.25)	0.98
Gender, Male	1.76 (0.90–3.47)	0.10		
ECOG PS ≥ 2	1.75 (0.91–3.36)	0.096	2.19 (1.09–4.42)	0.028
BMI ≥ 25	0.77 (0.27–2.17)	0.62	0.66 (0.22–2.01)	0.47
Current or former smoker	1.21 (0.47–3.11)	0.69	1.23 (0.46–3.30)	0.68
Histologic type, Sq	1.68 (0.87–3.25)	0.12		
PD-L1 TPS ≥ 50%	0.48 (0.25–0.93)	0.030	0.45 (0.23–0.88)	0.020
Goddard score ≥ 1	0.41 (0.21–0.80)	0.009	0.36 (0.18–0.71)	0.004
Treatment line ≥ 2	1.98 (1.03–3.82)	0.041		

*Sq* squamous cell carcinoma, *ECOG PS* Eastern Cooperative Oncology Group performance status, *BMI* body mass index, *TPS* tumor proportion score.

**Table 4:** Univariate and multivariate analysis of risk factors related to overall survival.

Variables	Univariate analysis		Multivariate analysis	
	HR (95% CI)	P-value	HR (95% CI)	P-value
Age ≥ 75 years	0.69 (0.26–1.81)	0.45	0.46 (0.16–1.29)	0.14
Gender, Male	1.57 (0.69–3.56)	0.28		
ECOG PS ≥ 2	1.78 (0.82–3.86)	0.15	3.62 (1.42–9.19)	0.007
BMI ≥ 25	0.42 (0.16–1.11)	0.079	1.00 (0.32–3.09)	> 0.99
Current or former smoker	2.43 (0.57–10.26)	0.23	2.78 (0.62–12.48)	0.18
Histologic type, Sq	1.59 (0.72–3.51)	0.25		
PD-L1 TPS ≥ 50%	0.35 (0.16–0.75)	0.007	0.26 (0.11–0.59)	0.001
Goddard score ≥ 1	0.52 (0.24–1.13)	0.099	0.30 (0.13–0.70)	0.005
Treatment line ≥ 2	1.79 (0.83–3.87)	0.14		

*Sq* squamous cell carcinoma, *ECOG PS* Eastern Cooperative Oncology Group performance status, *BMI* body mass index, *TPS* tumor proportion score.

## Discussion

This study investigated the impact of emphysema on the efficacy of PD-1/PD-L1 inhibitor therapy in clinical practice. Our results showed that emphysema (GS ≥ 1) evaluated by semiautomatic image analysis was significantly associated with better treatment outcomes for immunotherapy, while smoking status was not. The degree of emphysema, be it mild (GS 1–7) or moderate (GS ≥ 8), did not affect the efficacy of the ICIs. While one previous study has reported a relationship between GS and the efficacy of ICIs^5^, our study is novel because we excluded patients with oncogenic driver mutations and used semiautomatic imaging software to evaluate emphysema. Our chosen method of using image analysis software is accepted as more objective, reproducible, and technically easy.

Our study demonstrated that emphysema was correlated with better efficiency of ICIs compared to smoking status. Among the components of chronic obstructive pulmonary disease (COPD), which include emphysema and airway disease, emphysema enhances the therapeutic effect of PD-L1 blockade by exhausting the early cytotoxic CD8+ T cells^6^. Dendric cells exposed to an emphysema tumor microenvironment downregulate MHC class II and costimulatory molecules and upregulate PD-L1/IDO^6^. Our results are consistent with this mechanism, suggesting that the morphological change induced by smoking plays a more important role in improving the sensitivity of ICI than smoking itself.

Previous reports that investigated the relationship between COPD and therapeutic effects of ICI^7,8^ focused on airflow obstruction and not emphysema, while we focused on emphysema and did not evaluate airway components because of the lack of data on pulmonary function tests. At present, there are no data on which of these factors affect the microenvironments of lung cancer or whether both factors are related.

We measured the GS using semiautomatic software and used it to predict the therapeutic effect of ICIs. Several previous studies have visually assessed the association between emphysema and lung cancer mortality^9,10^. One report also demonstrated that visually assessing the presence of emphysema was suitable for predicting the risk of lung cancer^4^. However, there is evidence of the superior efficacy of automatically evaluating emphysema^11^, wherein a quantitative software-based method is used to detect panlobular emphysema, an important indicator of lung cancer mortality. Our results supported the efficiency of this semiautomatic method, and we believe that the method had high validity and was able to reproduce the same result and remove bias among different institutions.

Our results also indicated poor survival in patients with high PD-L1 status and without emphysema than in those with both emphysema and high PD-L1 status. Interestingly, even among patients with high PD-L1 expression, the treatment efficacy varied widely depending on the presence or absence of emphysema. Previous studies have shown that systemic inflammation, such as cachexia and sarcopenia, is associated with poor outcomes with immunotherapy^12^, even in patients with high PD-L1 expression^13^; however, local inflammation in the lungs may contribute to better outcomes with immunotherapy. To date, there are no data to explain these phenomena. High BMI has also been reported to be associated with ICI efficacy ^14^, but no association was found in our study. This may be partly due to the small sample size, but it was also possible that body composition may be more important to ICI efficacy than body weight; severe muscle depletion was reportedly associated with lower survival in obese patients with cancer ^15,16^. We believe it may be valuable to investigate the mechanisms underlying these effects and uncover the paradox of treatment failure even in patients with high PD-L1 expression or high BMI.

The present study has some limitations. First, this was a retrospective study conducted at a single center with a small sample size, precluding definite conclusions. Second, not all patients had the same imaging conditions in their CT scans, and different imaging conditions may have affected the evaluation of emphysema. Third, the follow-up time of 11.0 months in the present study may not be sufficient to evaluate overall survival. Finally, we could not assess the factor of airflow obstruction because of the lack of data on pulmonary function tests. Therefore, our findings should be regarded as hypothesis-generating for future studies.

In conclusion, we found that GS ≥ 1 evaluated by semiautomatic imaging analysis was associated with better treatment outcomes in patients who received monotherapy with ICIs. Thus, we believe that quantitative evaluation of emphysema can be used as an objective parameter to predict the therapeutic effects of ICIs in patients with advanced lung cancer. Further studies are warranted to validate our findings and resolve the method behind the underlying mechanisms of emphysema and ICIs.

## Materials and methods

### Study design and patients

We retrospectively analyzed patients with advanced NSCLC who received monotherapy with anti-programmed cell death-1/ligand-1 (PD-1/PD-L1) in our hospital from February 2018 to July 2019. Our exclusion criteria comprised patients who were receiving molecular targeted therapy, concurrently receiving any other anticancer therapy, or those who did not undergo chest CT within one month of starting treatment. We collected data on the patients’ backgrounds from their medical records, including age, sex, BMI, ECOG PS, smoking status, the number of patients evaluated by high-resolution computed tomography (HRCT), histology, PD-L1 tumor proportion score (TPS), clinical stage, treatment regimen and line, and baseline laboratory findings (< 2 weeks within starting treatment), which included data on C-reactive protein (CRP), serum albumin (ALB), and the neutrophil-to-lymphocyte ratio (NLR). We used BMI </≥ 25 as the binomial cut-off for our univariate and multivariate analyses, as the previous report defined^14^. We determined the treatment response using the Response Evaluation Criteria in Solid Tumors version 1.1. We also postulated the dates of progression, death, or the last follow-up. The cut-off date for data collection was August 30, 2020. Our study was approved by the ethics committee of Osaka University Hospital (approval no: 20445). This study was conducted in accordance with the 1964 Declaration of Helsinki and amendments. In view of the retrospective nature of the study, written informed consent from the subjects was waived by the committee.

### CT assessment of emphysema

To evaluate pulmonary emphysema in both lungs, we performed chest CT just before introducing ICIs. We selected CT scans with 0.5–2.0 mm slice thickness; if not available, we selected CT scans with 5 mm slice thickness.

We semiautomatically measured the GS to perform a quantitative evaluation of pulmonary emphysema on CT scans that we selected using SYNAPSE VINCENT imaging analysis software (Fujifilm Medical, Tokyo, Japan). We divided the entire field of each lung into three segments according to the horizontal lines at the top of the aortic arch, carina, and diaphragm. Using a 5-point scale for each segment, we scored the percentage of low-attenuation areas according to the corresponding Goddard classification^17^. We considered low attenuation areas of less than − 950 HU to indicate emphysema. No emphysema scored 0 points; ≤ 25% emphysema, 1 point; ≤ 50% emphysema, 2 points; ≤ 75% emphysema, 3 points; and > 75%, 4 points.

**Figure 3:**
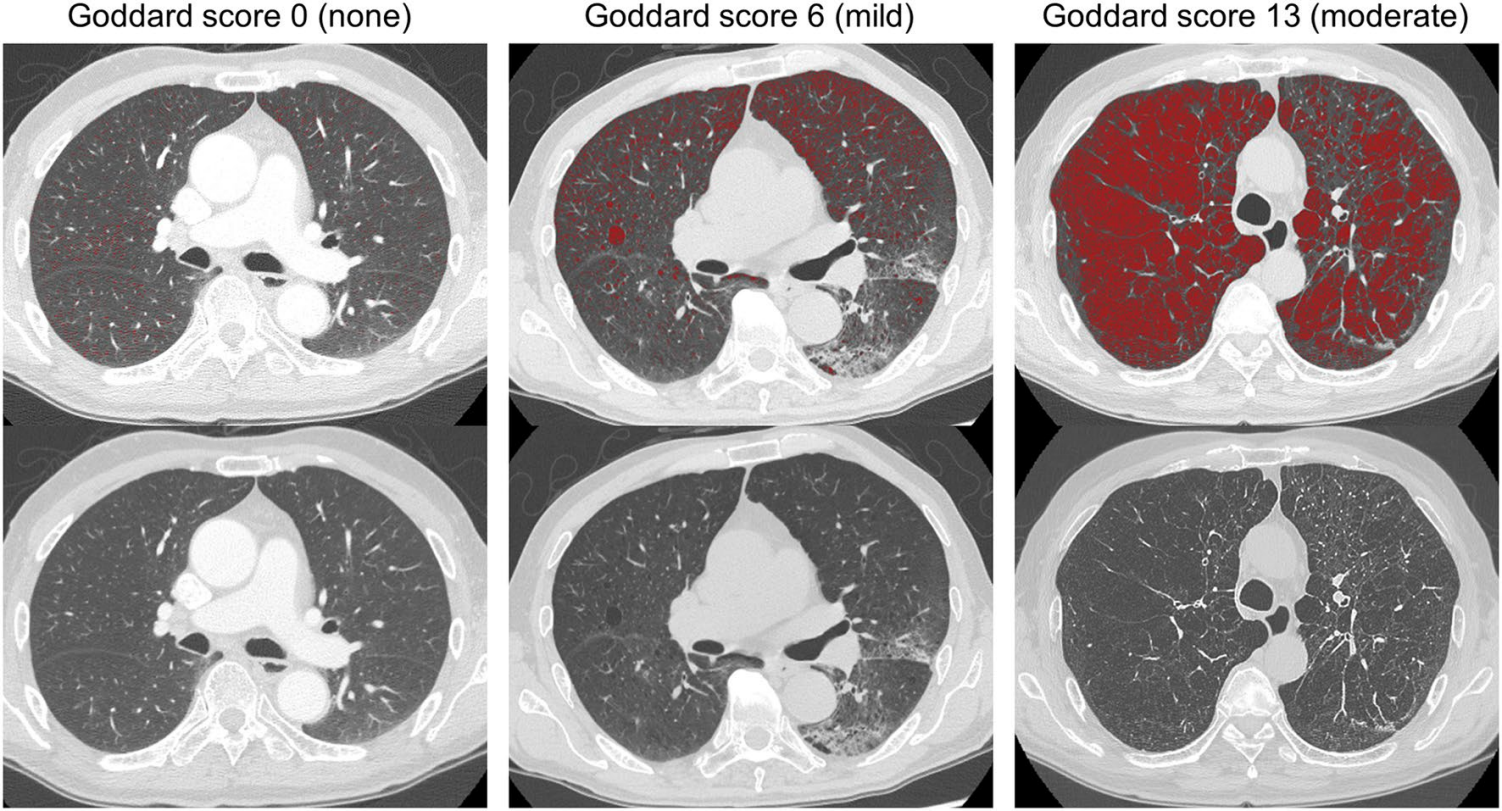
Representative computed tomography images showing the Goddard score using SYNAPSE VINCENT software (Fujifilm Medical, Tokyo, Japan).

We calculated the total points from all six segments for each patient as a personal Goddard score ranging from 0 to 24. Based on these CT findings, we divided all patients into two groups: GS 0 (non-emphysema group) and GS ≥ 1 (emphysema group). The emphysema group consisted of patients with mild (GS 1–7) and moderate emphysema (GS ≥ 8). Representative CT images obtained using the Goddard score are shown in Fig. 3.

### Statistical analysis

The clinical characteristics of both groups were compared using the Mann–Whitney U-test for continuous variables and Pearson’s Chi-square test for categorical variables. Survival curves were analyzed using the Kaplan–Meier method and log-rank test. Univariate and multivariate analyses were performed using the Cox proportional hazards model. Differences were considered statistically significant at a p-value of < 0.05. All statistical analyses were performed using R software (version 3.3.2).
